# Qualitative and Quantitative Characterization of Monosaccharide Components of* Salvia miltiorrhiza*, Liguspyragine Hydrochloride, and Glucose Injection

**DOI:** 10.1155/2017/9245620

**Published:** 2017-04-11

**Authors:** Fa-huan Ge, Xian-peng Ma, Jin-fang Ma, Chang-qiong Bi, Tian-ling Chen, Xiang-dong Zhang, Xue Xiao

**Affiliations:** ^1^School of Pharmaceutical Sciences, Sun Yat-sen University, Guangzhou 510006, China; ^2^Nansha Research Institute, Sun Yat-sen University, Guangzhou 511458, China; ^3^Guizhou Jingfeng Injection Co., Ltd., Guiyang 550018, China; ^4^Research Institute of Traditional Chinese Medicine, Guangdong Pharmaceutical University, Guangzhou 510006, China

## Abstract

*Salvia miltiorrhiza*, liguspyragine hydrochloride, and glucose injection (SLGI) was made of Salvia miltiorrhiza Bge., liguspyragine hydrochloride, glucose, and glycerin. There were many kinds of monosaccharide components in SLGI, which might be from the raw material and* Salvia miltiorrhiza* Bge. Separation was performed on a Phenomenex Luna C_18_ analytical column (250 mm × 4.6 mm i.d., 5 *μ*m, AccuStandard Inc., USA) at 30°C. The mobile phase consisted of two solvents: 0.1 mol/L phosphate-buffered saline (pH 6.7) (solvent A) and acetonitrile (solvent B) with gradient elution. The flow rate was maintained at 1.0 mL/min. Five kinds of monosaccharide components, glucose, D-mannose, L-rhamnose monohydrate, galactose, and xylose, were detected by precolumn derivatization HPLC, and their contents were compared with each other. And finally, concentrations of glucose in SLGI were determined and they were higher than the values of marked amount, which showed that one source of glucose might be from* Salvia miltiorrhiza* Bge. in SLGI. The average concentration of glucose was 5.18 g/100 mL, which was near the average value at 5.25 g/100 mL detected by ultraviolet spectrophotometry and also close to the marked amount (5.00 g/100 mL) on the label.

## 1. Introduction

In China, a large number of compound preparations are registered as chemical drugs; in spite of that they are from traditional Chinese medicine (TCM) and natural medicine, especially some injection. The levels of quality standard and quality control of compound preparations registered as chemical drugs are higher than Chinese medicine approval. However, the quality control system of TCM is not well built, especially the injections registered as chemical drugs from TCM and natural medicine. Compared to the classic chemical drugs, these injections are of various ingredients. Normally, the injections are from Chinese medicine extract with high purity as an intermediate, drug substance, medical supplements, and so forth. The classic example is* Salvia miltiorrhiza*, liguspyragine hydrochloride, and glucose injection (SLGI), which is made of* Salvia miltiorrhiza* Bge., liguspyragine hydrochloride, glucose, and glycerin. SLGI was used for occlusive ischemic cerebrovascular disease and other vascular diseases for that it can promote antiplatelet aggregation, expand coronary artery, reduce blood viscosity, speed up the flow rate of the red blood cells, and improve microcirculation against myocardial ischemia and myocardial infarction [1]. The quality control is carried out via quantification of one or several components from TCM.

However, only danshensu and liguspyragine hydrochloride are the quality indexes according to the quality standard, not considering the other components, such as glucose and salvianolic acid compositions. There were glucose, L-rhamnose monohydrate, xylose, D-mannose, galactose, and other carbohydrate compositions in* Salvia miltiorrhiza* Bge. So, sources of glucose in SLGI were from the raw material and* Salvia miltiorrhiza* Bge. through the study of production processes. And the above monosaccharides may be in SLGI [2].

Most of the literatures on the pharmacological action and clinical application of SLGI were studied. So far no studies reported on the determination of monosaccharide content. The common method used to determine the monosaccharide components is UV-Vis spectrophotometric method, which also determines the total amount of all monosaccharide components without selectivity. And in this research the qualitative identification and quantification of monosaccharide components are implemented by precolumn derivatization HPLC [3–7].

## 2. Materials and Methods

### 2.1. Materials and Reagents

The reference compounds of L-rhamnose monohydrate (BCBL0552V, 98%) and xylose (WXBB5741V, 98%) were purchased from Sigma-Aldrich Co., Ltd., and D-mannose (LOTD1429046, 98%) and 3-Methyl-1-phenyl-2-pyrazoline-5-one (PMP, 99%) were from Aladdin Co., Ltd., and glucose (LOT110833-201205, 98%) and galactose (LOT100226-201105, 98%) were purchased from the National Institute for Food and Drug control (Beijing, China).

HPLC-grade acetonitrile was purchased from Merck (Darmstadt, Germany) and formic acid (99.99%, HPLC) was obtained from Tianjin Fuyu Fine Chemical Co., Ltd. (Tianjin, China). High pure water (18.2 MΩ) was purchased from Wahaha Group Co., Ltd. (Hangzhou, China). Other reagents were of analytical grade. Ten batches of SLGI commercial products were supplied by Guizhou Jingfeng Injection Co., Ltd. (Guiyang, China).

### 2.2. Preparation of Standard Solutions and Sample Solutions

#### 2.2.1. Sample Solutions for HPLC-UV

The SLGI solutions were stored at 4°C. The solutions were brought to room temperature, diluted, and filtered through a 0.45 *μ*m membrane filter before analysis.

The samples of SLGI for HPLC-UV analysis were injected for HPLC-UV analysis.

#### 2.2.2. PMP Solution [8, 9]

PMP, 3-methyl-1-phenyl-2-pyrazolin-5-one, and monosaccharides produce monosaccharides-PMP monosaccharide derivatives under the condition of alkaline and quantitative condensation. 21.775 g of PMP was weighed, dissolved in 250 mL with methanol, and then filtered through a 0.45 *μ*m membrane filter before analysis.

#### 2.2.3. Standard Solutions

Each reference was accurately weighed, dissolved in deionized water, and diluted to the appropriate concentration. And the standard solutions were filtered through a 0.45 *μ*m membrane filter before analysis.

#### 2.2.4. Derivatization Method

The SLGI and standard solutions derivatization process was dealt with with the following the procedure. The SLGI sample was accurately drawn and mixed with 200 *μ*L of the sodium hydroxide solution (0.3 mol/L) and 160 *μ*L of PMP solution (0.5 mol/L), separately. Then the mixed solution was carried out upon a water bath and heated for 30 min and cooled down to room temperature. Next, 200 *μ*L of the hydrochloric acid solution (0.3 mol/L) was injected to neutralize the mixed solution. And then, 1,240 *μ*L of purified water and 2 mL of chloroform were added in the prior mixed solution and well mixed, which was centrifuged at 3,000 rpm for 5 minutes, discarding the chloroform layer to collect the water layer. After extraction for 3 times, the water layers were collected and filtered through a 0.45 *μ*m membrane filter before analysis.

### 2.3. HPLC Apparatus and Conditions

HPLC-UV analysis was performed using an UltiMate 3000 HPLC-UV system (Thermo Fisher Scientific, New York, USA) compressing a vacuum degasser, binary pump (SR-3000), autosampler (WPS-3000), thermostatted column compartment (TCC-3000), and a diode array detector (DAD-3000); scanning from 200 to 400 nm, the wavelength was then selected and fixed at 250 nm for qualitative and quantitative analysis [10], considering the variety of constituents in SLGI. Separation was performed on a Phenomenex Luna C_18_ analytical column (250 mm × 4.6 mm i.d., 5 *μ*m, AccuStandard Inc., USA) at 30°C. The mobile phase consisted of two solvents: 0.1 mol/L phosphate-buffered saline (pH 6.7) (solvent A) and acetonitrile (solvent B) with gradient elution (0–3 min, 13-13% B; 3–8 min, 13–16% B; 8–15 min, 16–23% B; 15–30 min, 23-23% B). Reequilibration duration was ten min between individual runs. The flow rate was maintained at 1.0 mL/min and 10 *μ*L of sample solution was injected in each run.

## 3. Results and Discussion

### 3.1. Optimization of HPLC Conditions [11]

**Figure 1:**
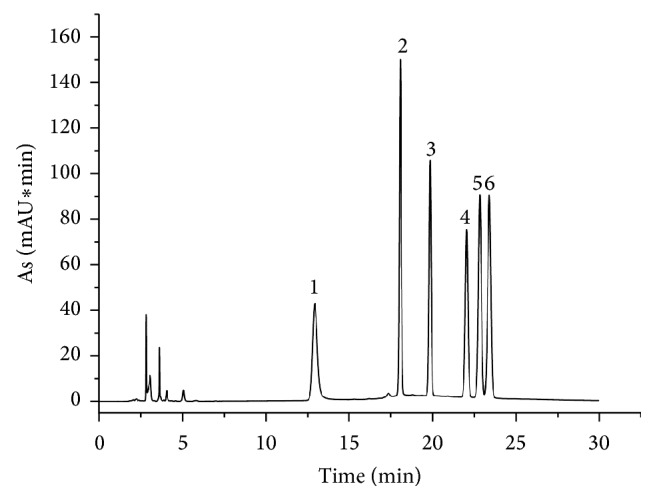
The chromatogram of monosaccharide of mixed standard solution (1: PMP; 2: D-mannose; 3: L-rhamnose monohydrate; 4: glucose; 5: galactose; 6: xylose).

**Figure 2:**
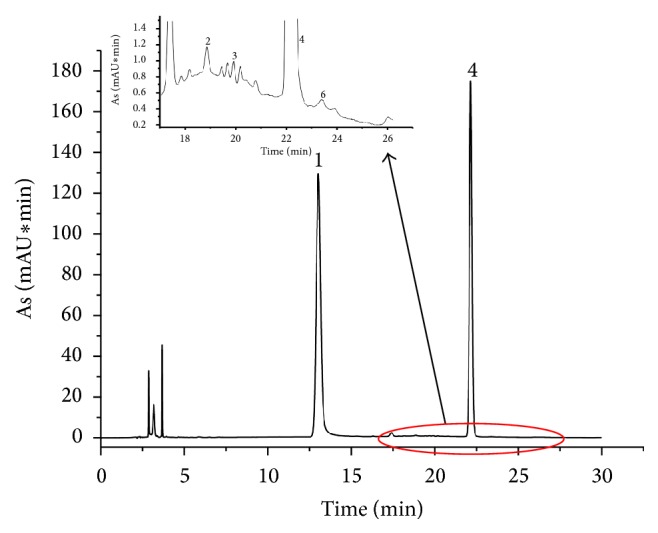
The typical chromatogram of monosaccharide of SLGI (1: PMP; 2: D-mannose; 3: L-rhamnose monohydrate; 4: glucose; 6: xylose).

Column types, mobile phase compositions, gradient elution procedure, flow rate of the mobile phase, and column temperature were optimized, respectively, to achieve good separation of as many peaks as possible within a short analysis time. Different mobile phase systems, such as acetonitrile/water, methanol/water, acetonitrile/0.1 mol/L ammonium acetate, and acetonitrile/0.1 mol/L phosphate-buffered saline (pH 6.7), were tested. Results showed that addition of acetonitrile/0.1 mol/L phosphate-buffered saline (pH 6.7) was more suitable to obtain better peak symmetry and stable baseline and/or to inhibit ionization of the acidic ingredients in SLGI. The applicability of the established analysis method to different HPLC instrument systems has been verified. The detection wavelength was selected with the use of a DAD detector. The detection wavelength for quantitative analysis was selected according to the maximum adsorption wavelengths of D-mannose, L-rhamnose monohydrate, glucose, galactose, and xylose at 250 nm, as shown in Figure 1. The typical chromatogram of SLGI was illustrated in Figure 2, in which there were D-mannose, L-rhamnose monohydrate, glucose, and xylose in SLGI.

### 3.2. Method Validation of Quantitative Analysis

The HPLC method was validated defining the linearity, limits of quantification, and detection, identification and quantification of the analyte, repeatability, precision, stability, and recovery. The work was performed under the validation guide according to the “0512 high performance liquid chromatography” in* Chinese Pharmacopoeia* Part IV (2015 edition).

#### 3.2.1. Calibration Curves, LODs, and LOQs

The calibration curve was plotted based on linear regression analysis of the integrated peak areas (*y*) versus concentrations (*x*, mg/mL) of glucose in the standard solution at six different concentrations. The regression equation was displayed as *y* = 64.806*x* − 0.803  8, correlation coefficient was 0.999 4, and linear range for the analysis of glucose was from 0.0313 mg/mL to 1.000 0 mg/mL. The LOD was calculated as the amount of the injected sample which gave a signal-to-noise ratio of 3 (S/N = 3 : 1), and the LOQ was calculated as the amount of the injected sample which gave a signal-to-noise ratio of 10 (S/N = 10 : 1).

#### 3.2.2. Precision, Repeatability, Stability, and Recovery

Intraday variation was chosen to determine the precision of the developed assay. The SLGI sample solutions were analyzed for six replicates within one day. Variation was expressed by the RSD, which was less than 1.0% (Table 1). Six different sample solutions prepared from the same sample were analyzed to confirm the repeatability of the developed assay. RSD values of the compound's contents were all less than 2.0%, which satisfied the criteria of quantitative analysis (Table 1). For the stability test, peak areas of glucose in sample solution, which was stored at room temperature in the dark, were analyzed at 0 h, 4 h, 8 h, 16 h, and 24 h. RSD value of the content was less than 1.0% (Table 1). These results expressed the view that it was feasible to analyze the samples within one day. The ratio between amounts determined and spiked was considered to be able to display the accuracy of the method. Known amounts (low, medium, and high) of the glucose reference were spiked into samples and then prepared as test solutions. The determination was performed in triplicate, and the average recoveries and RSD were calculated. The developed method had good accuracy with the overall recovery at 102.9%, with the RSD ranging at 3.36% (Table 1). These results indicate that the HPLC-UV method is precise, accurate, and sensitive for the quantitative determination of glucose in SLGI samples.

### 3.3. Chemical Profiling and Quantitation

Chemical profiling and assay of 10 different commercial batches of SLGI were performed. As for the quantitative analysis, glucose was invoked as the marker component in the enacted quality standard of SLGI recorded in the national standard for Chinese patent medicine. The developed determination method was subsequently applied to determination of glucose in 10 different commercial batches of SLGI. The results are presented in Table 2. The content of glucose varied bittily, and the RSD (%) of contents was 2.20.

There was no obvious difference about the concentrations of glucose in 10 batches of SLGI. And the average concentration of glucose was 5.18 g/100 mL, which was near the average value at 5.25 g/100 mL detected by ultraviolet spectrophotometry and also close to the marked amount (5.00 g/100 mL) on the label.

### 3.4. Discussion

As shown from Figures 1 and 2, there were D-mannose, L-rhamnose, glucose, and xylose in SLGI. But the peak areas of D-mannose, L-rhamnose monohydrate, and xylose were much smaller than the peak area of glucose (Table 3). That is to say, the main monosaccharide was glucose in SLGI. And the ratio of the peak area of glucose to the total peak area of monosaccharide (G/T) was higher than 98%. So the determination of glucose could characterize the total content of monosaccharide in SLGI.

## 4. Conclusion

Precolumn derivatization HPLC method, as shown in the paper, could clearly show the content of each component rather than the total amount of all monosaccharide components without selectivity, which could be more intuitive for evaluation of the quality of the SLGI attributes. And many studies on monosaccharide components, amino acids, salvianolic acids, and other substances are carried out to reveal the material basis of SLGI. In current quality standard of SLGI, however, there is no mandatory requirement of the above components according to* The Standard Issued by the Ministry of Health* (number WS-10001-(HD-1136)-2002). As a valuable supplement, the method may provide a new way to improve the quality control of compound preparations, such as TCM compound preparations.

## Figures and Tables

**Table 1:** Precision, repeatability, stability, and recovery of glucose in in SLGI (*n* = 6).

Number	Precision peak area (mAU·min)	Repeatability concentrations (g/100 mL)	Stability peak area (mAU·min)	Recovery (%)
1	84.9381	5.0235	34.3784	95.9
2	84.9562	5.0191	34.0784	103.9
3	84.9570	5.0149	33.9660	104.8
4	84.8971	5.1066	33.8164	104.2
5	84.8200	5.1574	33.9377	103.9
6	84.8356	5.0779	33.8485	104.7
Average	84.9007	5.0666	34.0042	102.9
RSD (%)	0.07	1.14	0.60	3.36

**Table 2:** The results of determination of glucose in SLGI.

Number	Concentration (mg/mL)	Concentration (g/100 mL)
20140413	54.29	5.43
20140414	51.74	5.17
20140415	51.52	5.15
20140416	51.67	5.17
20140417	52.36	5.24
20140418	51.41	5.14
20130517	52.77	5.28
20130724	50.11	5.01
20131249	51.46	5.15
20130135	50.78	5.08
Average	51.81	5.18
RSD (%)	2.20	2.20

**Table 3:** The peak areas of monosaccharides in SLGI (As).

Batch	Number	D-Mannose	L-Rhamnose	Glucose	Total peak area	G/T (%)
201310135	1	0.271 2	0.0333	31.75	32.05	99.06
	2	0.273 3	0.0338	31.72	32.03	99.03
	3	0.274 7	0.0328	31.69	32.00	99.03
	4	0.277 5	0.0195	32.29	32.59	99.08
	5	0.273 9	0.0290	32.62	32.92	99.09
	6	0.362 2	0.0169	32.10	32.48	98.83
	Average	0.288 8	0.0276	32.03	32.35	99.01
